# Brivaracetam-Associated Rhabdomyolysis Requiring Renal Replacement Therapy: A Case Report and Review of the Literature

**DOI:** 10.7759/cureus.58183

**Published:** 2024-04-13

**Authors:** Khaled Alok, Amanda Lin, Celine Rahman Dematteo, Husain Abrar, Zachary Levy

**Affiliations:** 1 Neurocritical Care, Northwell Health, Manhasset, USA; 2 Pharmacy, Neurocritical Care, Northwell Health, Manhassett, USA; 3 Internal Medicine, Northwell Health, Manhasset, USA

**Keywords:** cerebral cavernoma, status epilepticus (se), adverse effects, antiseizure medications, rhabdomyolysis, brivaracetam

## Abstract

Rhabdomyolysis is a rare adverse reaction that has a previously established association with levetiracetam use, which selectively binds the synaptic vesicle glycoprotein 2A (SV2A). Its structural analogue, brivaracetam, is a new third-generation antiseizure medication that has a higher affinity for SV2A, and current data suggests it provides a more favorable adverse event profile. Here, however, we report a case of rhabdomyolysis requiring dialysis in which serum creatine kinase level increased rapidly for several days until brivaracetam was discontinued. The delayed creatine kinase peak, rapid decline upon discontinuation of brivaracetam, and prior association of rhabdomyolysis with levetiracetam strongly suggest a causal relationship. To date, there are three reported cases of brivaracetam-associated rhabdomyolysis in the food and drugs administration adverse event reporting system (FAERS). Despite its favorable side effects profile, the use of brivaracetam may be associated with life-threatening rhabdomyolysis.

## Introduction

Adverse drug reactions are among the leading causes of poor adherence to antiseizure medication (ASM) therapy, contributing to higher epilepsy-related morbidity and increased frequency of unscheduled care [1]. Second and third-generation ASMs provide a favorable safety profile, decreased drug-drug interactions, and a lower risk of teratogenicity [2]. Post-marketing surveillance, however, has identified an association between newer ASMs and several rare yet life-threatening adverse reactions. For example, the Food and Drug Administration (FDA) recently issued a warning that levetiracetam and clobazam can precipitate lethal cases of Drug Reaction with Eosinophilia and Systemic Symptoms (DRESS) syndrome [3].

Rhabdomyolysis is another serious adverse reaction that is linked to several newer-generation ASMs, including levetiracetam, pregabalin, lamotrigine, gabapentin, topiramate, oxcarbazepine and lacosamide [4,5]. Of the ASMs, levetiracetam has the largest number of published case reports describing its association with rhabdomyolysis [4]. A query in the FDA adverse events reporting system (FAERS) retrieved 923 reports of levetiracetam-associated rhabdomyolysis, 39 of which resulted in patient mortality [6].

The third-generation ASM brivaracetam is a structural analogue of levetiracetam that has a 15-30 times greater affinity to their common ligand, synaptic vesicle 2A protein (SV2a). In addition, brivaracetam offers potentially better tolerability and reduced psychiatric side effects [7]. However, the potential for brivaracetam to cause rhabdomyolysis has yet to be established. Here, we report a case of brivaracetam-associated severe rhabdomyolysis. The structural similarity to levetiracetam prompted discontinuation of the medication, with rapid normalization of all serum biomarkers.

## Case presentation

A 22-year-old morbidly obese African-American male (BMI 40.7 kg/m2, weight: 136 kg, height 182.9 cm) with no previous medical history presented to the emergency department (ED) following a generalized tonic-clonic seizure (GTCS) that aborted spontaneously within two minutes. During the initial ED evaluation, the patient was awake but lethargic, and complained of sore thighs and shoulders. While in the ED, he developed another GTCS that resolved spontaneously in less than a minute. However, the patient did not return to his baseline neurological exam and was assumed to be in status epilepticus. As such, he was subsequently intubated, loaded with intravenous 4000 mg of levetiracetam (30 mg/kg), started on continuous infusions of propofol and midazolam, and transferred to our neurocritical care unit.

Upon arrival, a continuous electroencephalogram (cEGG) showed a burst-suppression background with >90% suppression without epileptiform discharges. The patient was weaned off all anesthetics over the next 48 hours and was successfully extubated. Neurological examination revealed proximal muscle weakness in the upper and lower extremities that gradually improved over the following days. Contrast-enhanced computed tomography revealed a hemorrhagic lesion in the right gyrus rectus with surrounding edema.

**Figure 1:**
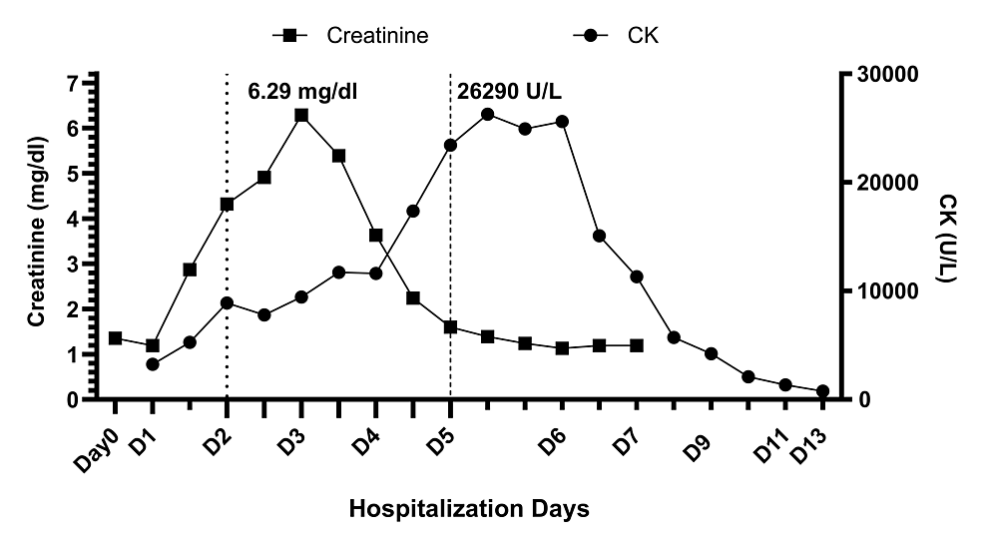
Timeline of serum creatinine and creatine kinase (CK) levels The figure represents the serum creatinine and CK levels over 13 days in the hospital. Day 0: The patient was started on levetiracetam. On day 2 the patient underwent one session of hemodialysis for acute kidney injury, and levetiracetam was switched to brivaracetam. On Day 5 brivaracetam was stopped and replaced with lacosamide. Creatinine peaked at 6.29 mg/dl on Day 3, however, CK kept rising to a peak of 26290 U/L on Day 5. Note the sharp decline in CK after 24 hours of the last dose of brivaracetam.

On initial presentation, serum workup revealed hyperkalemia and rapidly rising creatinine levels. Creatine kinase (CK), serum lactate dehydrogenase (LDH), and aspartate aminotransferase (AST) were all elevated. All findings were consistent with intrinsic acute renal injury (AKI) secondary to rhabdomyolysis and contrast nephropathy. The glomerular filtration rate (GFR) dropped from a baseline of 76 to a nadir of 12 mL/min/1.73 m^2^. One session of hemodialysis was required on day 2, as the patient’s renal function returned gradually to baseline over several days of treatment with high-rate crystalloid infusion and diuresis. Figure 1 summarizes the trend of serum creatinine and creatine kinase levels during the patient’s hospitalization.

Given the patient’s worsening renal function, he was switched on day 2 from intravenous levetiracetam to intravenous 100mg of brivaracetam every 12 hours, since the latter does not require renal dosing adjustment. Nonetheless, the upward trend in serum CK continued despite the rapid improvement in renal function. The patient was clinically stable, reporting near resolution of the initial shoulder and thigh pain. Antinuclear antibodies, mitochondrial antibodies, SSA-52 and SSA-60 IgG antibodies, anti-ribonuclear protein antibodies, and smooth muscle antibodies were all within normal limits.

Knowing the reported association between levetiracetam and rhabdomyolysis, and the structural similarities between brivaracetam and levetiracetam, brivaracetam was considered the possible precipitating factor for the persistent rhabdomyolysis. The patient was transitioned on day 5 from brivaracetam to lacosamide. CK level peaked on day 5 at 26290 U/L (normal range 30-200 U/L) then dropped sharply, six days after the index seizure and 24 hours after the last dose of brivaracetam (Figure 1).

The right gyrus rectus lesion was found to be, on subsequent magnetic resonance imaging, a cavernous malformation that was surgically resected 2 weeks later. 

## Discussion

Postictal elevation of serum CK following a seizure episode is considered a specific marker to diagnose GTCS [8]. The upper normal limit of serum CK varies with age, gender, muscle mass, and physical activity. However, an increase in serum CK as little as 15 U/L is considered predictive of an epileptic seizure [9]. A recent meta-analysis showed a 56.5% sensitivity for elevated CK on day 2 following an epileptic event, with mean values of 114 U/L and 213 U/L on day 1 and day 2, respectively [10]. Our patient’s CK level on day 2 was 8890 U/L, and it continued to increase exponentially until brivaracetam was discontinued. Despite the patient’s large muscle mass, this extreme and prolonged rise in serum CK cannot be attributed to the patient’s presenting GTCS alone.

Brivaracetam is a 4-n-propyl analogue of levetiracetam, and the two medications share many similarities in their pharmacodynamics [11]. A review of the FAERS database revealed three reported cases of brivaracetam-associated rhabdomyolysis, including one mortality [6]. To our knowledge, this is the first published case report on the matter. On the other hand, levetiracetam-associated elevated CK serum levels have been reported in various age groups, in postictal as well as interictal contexts, often within 12-36 hours of initiating treatment and appearing to be completely reversible with levetiracetam discontinuation [12].

The mechanism of this adverse reaction is unknown. One study suggested that since levetiracetam is the biologically active enantiomeric form of etiracetam, it can behave as an acetylcholine agonist. This theory stipulates that levetiracetam can potentiate neuromuscular transmission, resulting in a high-stress level with energy depletion in the striated muscle cells [13]. It is reasonable to hypothesize that this mechanism may apply to brivaracetam as well. 

## Conclusions

Similar to levetiracetam, the use of brivaracetam seems to be associated with rhabdomyolysis. Further research is required to identify any underlying factors that predispose a minority of patients receiving levetiracetam or brivaracetam to develop this adverse effect. In the meantime, awareness of the potential for brivaracetam to induce this potentially life-threatening adverse reaction is essential for early recognition and prompt management. 
